# A Miniature Optical Force Dual-Axis Accelerometer Based on Laser Diodes and Small Particles Cavities

**DOI:** 10.3390/mi12111375

**Published:** 2021-11-04

**Authors:** Junji Pu, Kai Zeng, Yulie Wu, Dingbang Xiao

**Affiliations:** College of Intelligent Sciences, National University of Defense and Technology, Changsha 410073, China; pujunji21@nudt.edu.cn (J.P.); zengkai19@nudt.edu.cn (K.Z.); dingbangxiao@nudt.edu.cn (D.X.)

**Keywords:** optical force acceleration, laser diode, elliptical Gaussian beam, MEMS particles cavity

## Abstract

In recent years, the optical accelerometer based on the optical trapping force effect has gradually attracted the attention of researchers for its high sensitivity and high measurement accuracy. However, due to its large size and the complexity of optical path adjustment, the optical force accelerometers reported are only suitable for the laboratory environment up to now. In this paper, a miniature optical force dual-axis accelerometer based on the miniature optical system and a particles cavity which is prepared by Micro-Electro-Mechanical Systems (MEMS) technology is proposed. The overall system of the miniature optical levitation including the miniature optical system and MEMS particles cavity is a cylindrical structure with a diameter of about 10 mm and a height of 33 mm (Φ 10 mm × 33 mm). Moreover, the size of this accelerometer is 200 mm × 100 mm × 100 mm. Due to the selected light source being a laser diode light source with elliptical distribution, it is sensitive to the external acceleration in both the long axis and the short axis. This accelerometer achieves a measurement range of ±0.17 g–±0.26 g and measurement resolution of 0.49 mg and 1.88 mg. The result shows that the short-term zero-bias stability of the two orthogonal axes of the optical force accelerometer is 4.4 mg and 9.2 mg, respectively. The main conclusion that can be drawn is that this optical force accelerometer could provide an effective solution for measuring acceleration with an optical force effect for compact engineering devices.

## 1. Introduction

In the past several decades, the accelerometer has played an important role in the field of measuring the linear acceleration of moving objects in real-time. According to different measuring principles and structure forms, the traditional accelerometers can be divided into electrostatic accelerometers, Micro-Electro-Mechanical System (MEMS) accelerometers, and flexible pendulous accelerometers [1,2]. Based on their performance indicators, different kinds of accelerometers have been widely used in civilian and military fields, including unmanned driving, weapon guidance, and other fields. In recent years, to improve the accuracy of navigation and positioning, high-precision accelerometers have attracted more and more attention and research. Among them, the accelerometer based on an optical-levitated particle has a high mechanical resolution, high measurement accuracy, and it can isolate the external thermal environment noise [3,4,5]. Therefore, it is widely considered to be a new type of accelerometer with high precision potential.

The basic principle of optical levitation technology is to focus the laser with a high numerical aperture (NA) lens or objective lens so that the micro/nanoparticles near the focus are subjected to the action of the optical trap force. When the optical trap force received by the particle is balanced with its gravity and external environmental resistance, the particle will be stably trapped in the optical trap [6]. The pioneering fundamental work of optical levitation technology was presented by Ashkin et al. [7]. Optical levitation technology has taken many forms since its birth [8,9,10,11]. The various forms of optical levitation technology enable it to meet the different research purposes of many disciplines, such as basic physics [12], medical diagnosis [13], high-precision measurement, and so on. Especially in the field of precision measurement, many research institutions have conducted in-depth research on optical force-sensing systems in recent years, including the measurement of force, torque, and acceleration [14,15,16,17,18]. Butts et al. [19] developed one optical force accelerometer based on optical levitation technology, which can preliminarily realize the measurement resolution of 119 μg/ $\sqrt{\mathrm{Hz}}$ within operating frequencies of 200–300 Hz. Then they improved both the resolution and zero-bias stability by removing the structure of the light beam expander [20]. Moreover, Monteiro [21] obtained a sensitivity of 0.4 μg/ $\sqrt{\mathrm{Hz}}$ for a 12-ng sphere in a high vacuum that occurs at frequencies ranging from 10 to 200 Hz. Rider et al. [22] presented a technique to levitate and measure the three-dimensional position of one 4.8 μm-diameter dielectric sphere with heterodyne detection. The result shows that the acceleration noise can reach 7.5 μg/ $\sqrt{\mathrm{Hz}}$ in the 10 to 100 Hz band.

**Figure 1:**
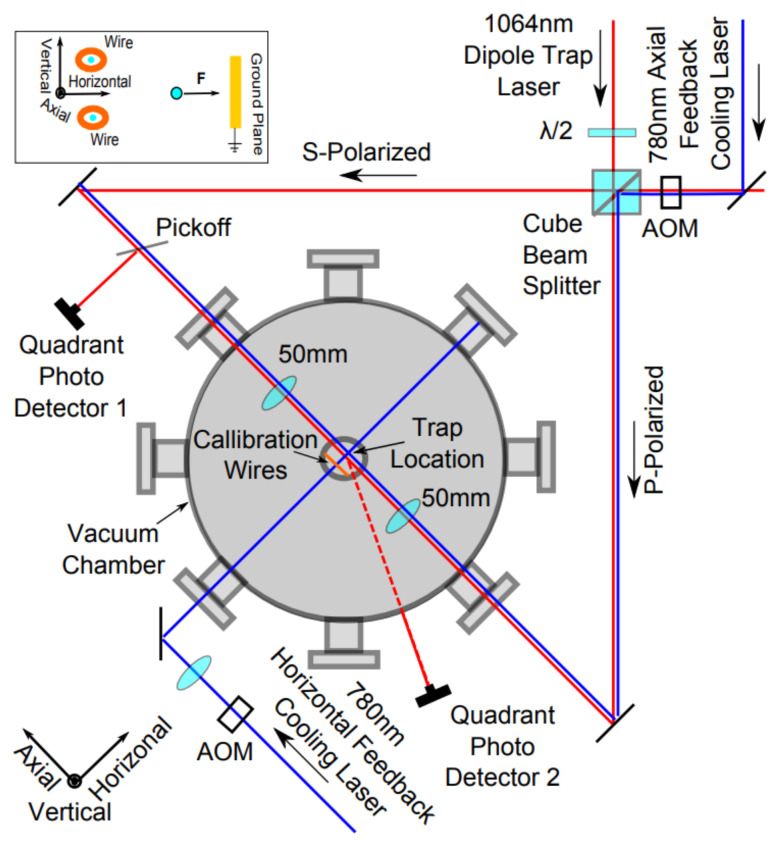
Optical path of the optical accelerometer from the group of Alexander et al. [23].

However, due to the large volume of the traditional desktop laser and vacuum chamber [23,24,25,26], as shown in Figure 1, the optical force-sensing systems mentioned above are only suitable for high-precision measurement in the laboratory environment rather than compact engineering devices.

This paper considers the miniaturization of the optical force accelerometer as the main subject of its study. A new form of miniature optical force accelerometers is proposed in our work. The whole accelerometer consists of an optical levitation system and a signal detection system. To decrease the volume of the levitation system as much as possible, the package volume of the optical system including one laser diode and a pair of lenses is designed to be a cylindrical construction with a diameter of about 8 mm and a height of 30 mm (Φ 8 mm × 30 mm). Under the demand of low measurement accuracy, a vacuum chamber in large volume is avoided. A MEMS cavity is finally determined to be the optimal structure to further reduce the packaging volume. The signal detection system consists of one four-quadrant photoelectric detector (QPD) and other beam shaping devices. The entire package volume of the accelerometer is 200 mm × 100 mm × 100 mm. Due to the elliptical light field distribution of the laser diode, the accelerometer can be sensitive to the external acceleration along the long axis and short axis of the optical field. To measure the stability of this system in an hour without external input, the whole device is fixed on a rotating platform that can rotate around two axes. Results show that the short-term zero-bias stability of the two orthogonal axes of the optical force accelerometer is 4.4 mg and 9.2 mg respectively. It suggests that this miniature optical force dual-axis accelerometer owns the potential to be a new form of accelerometer which may be taken advantage of in the field of inertial navigation.

## 2. Principle and Design of the Miniature Optical Levitation System

### 2.1. Principle of the Optical Force Dual-Axis Accelerometer

**Figure 2:**
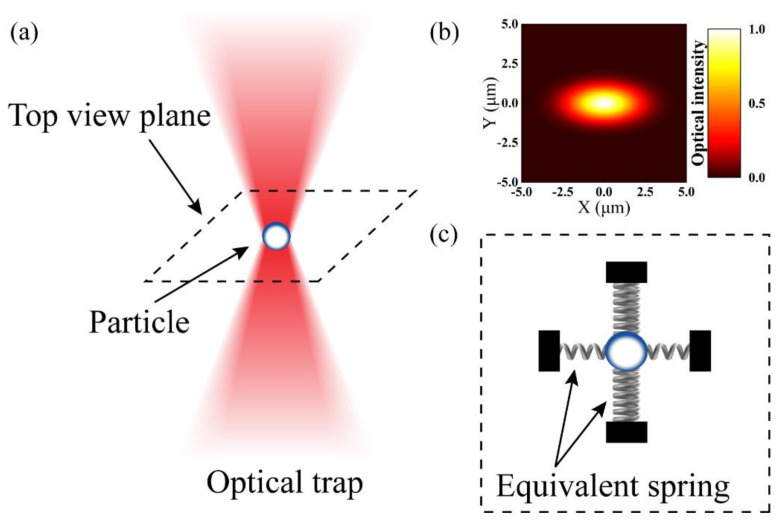
Schematic diagram of the optical force dual-axis accelerometer. (**a**) A single-beam optical trap used to levitate a particle; (**b**) Optical field distribution at the focus plane of one laser diode; (**c**) Equivalent spring oscillator model at the focal plane.

Due to the optical field distribution of a laser diode is not a standard gaussian distribution, it is necessary to analyze the principle of this accelerometer. As shown in Figure 2a, one particle is affected by a focused laser. When the stiffness of the optical trap is large enough, it will be stably trapped until the optical trap force is balanced to its gravity and other environmental forces such as intermolecular force and viscosity resistance. If there is an external disturbance, the particle will deviate from its stable position. At this time, the optical trap force will force the particle to return to its original position.

However, as can be seen from the simulation results in Figure 2b, the optical field distribution of the laser diode is more like an elliptical Gaussian beam rather than a standard gaussian beam. This leads to the nonuniformity of the optical trap stiffness in the circumferential direction. The stiffness of the ellipse Gaussian beam along the short axis is the largest and the stiffness along the long axis is the smallest. The equivalent spring oscillator model of the system at the focal plane is shown in Figure 2c.

When the same acceleration is input externally, the offset of the particle in two directions is not the same. It is also the reason why this optical force accelerometer has two sensitive axes.

The structure of the miniature optical levitation system determines the overall size of the optical force accelerometer to a great extent. Finally, we determined an optical levitation system with a single laser diode and a pair of lenses as the optical system and a MEMS particles chamber as the main body.

### 2.2. Design and Construction of the Optical System

**Figure 3:**
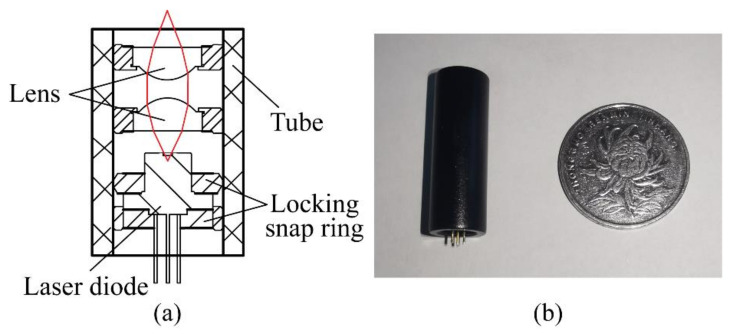
Optical system. (**a**) Structural drawing, the red line is the focused optical path; (**b**) Photograph of the optical system.

As we know, the divergence angles of the laser diode in two orthogonal axes range from 10° to 60°, which are too large to form an optical field with a strong gradient. Hence the size and numerical aperture (NA) of lenses are needed to be considered adequately. Finally, we decide to choose a pair of aspherical lenses with a diameter of 6 mm, NA of 0.28, and focal length of 7 mm, which are placed opposite. To acquire a larger optical trap stiffness in the air environment and avoid excessive heat accumulation, the power and wavelength of the laser diode are identified as 150 mW and 808 nm. After the entire optical system is assembled, the package volume of the system is Φ 8 mm × 30 mm. The assembly drawing and photograph of the optical system are shown in Figure 3.

### 2.3. Design and Construction of the MEMS Particles Cavity

At present, almost all of the reported optical force accelerometers adopt a vacuum cavity with large volume and high intensity to reduce the environmental noise, to realize a high precision measurement of acceleration. However, the vacuum cavity mentioned above is not suitable for some occasions where low accuracy is required, such as a tactical level in the electronics and automotive industry. Hence, we identify this MEMS cavity structure where particles are put in the atmospheric environment.

**Figure 4:**
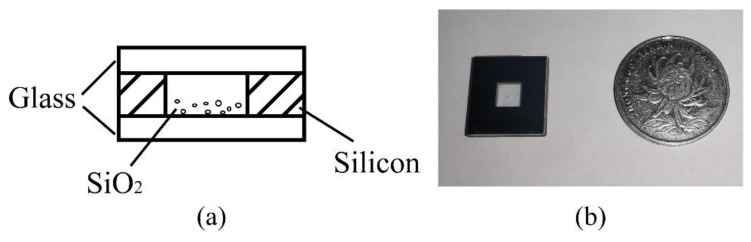
MEMS particles cavity. (**a**) Structural drawing; (**b**) Photograph of the MEMS particles cavity.

The whole chamber adopts a “glass-silica-glass” sandwich structure, and its schematic diagram is shown in Figure 4.

Due to the roughness of the cavity interior wall having little effect on the optical trap stiffness and measurement accuracy of the acceleration, ultraviolet nanosecond laser scribing is chosen as the chamber forming process rather than the time-consuming etching process. In order to ensure the success rate of the anodic bonding process, the silicon wafer and glass wafer need to be soaked and cleaned with deionized water and acetone solution. After ultrasonic cleaning, dry the silicon wafer and glass wafer with dry nitrogen. When the silicon substrate is prepared, its lower surface is bonded to one piece of glass by a one-sided anodic bonding process. After a few particles are placed on the bottom glass surface with one tweezer, the upper surface of the silicon wafer is bonded to another piece of glass by using the same process again. The temperature and voltage of the anode bonding process are set at 340 °C and 800 V. It is important to note that the thickness of the wafer must be taken into account to ensure that particles do not be thrown and adhere to the upper surface during excitation. After considering the light transmittance of glass, we chose the glass and silicon wafer with a thickness of 300 μm and 500 μm. Due to the thick silicon wafer, we adopt double-sided processing in order to quickly realize the above scribing process. The entire volume of the MEMS particles cavity is 10 mm × 10 mm × 1.1 mm.

### 2.4. Construction of the Miniature Optical Levitation System

**Figure 5:**
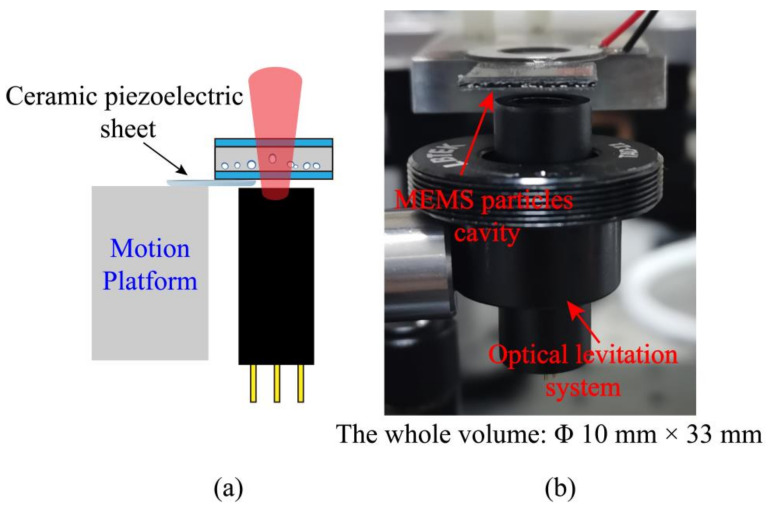
Miniature optical levitation system. (**a**) Overall schematic diagram; (**b**) Photograph of the miniature optical levitation system.

The lower surface of the MEMS particles cavity is bonded to a ceramic piezoelectric sheet, which is fixed on a triaxial displacement platform. The optical system is located directly below the MEMS particles cavity, to realize a vertical miniature optical levitation system with a single optical trap. The miniature optical levitation system is shown in Figure 5. The entire volume of it is Φ 10 mm × 33 mm.

## 3. Construction of the Optical Force Accelerometer and Test System

**Figure 6:**
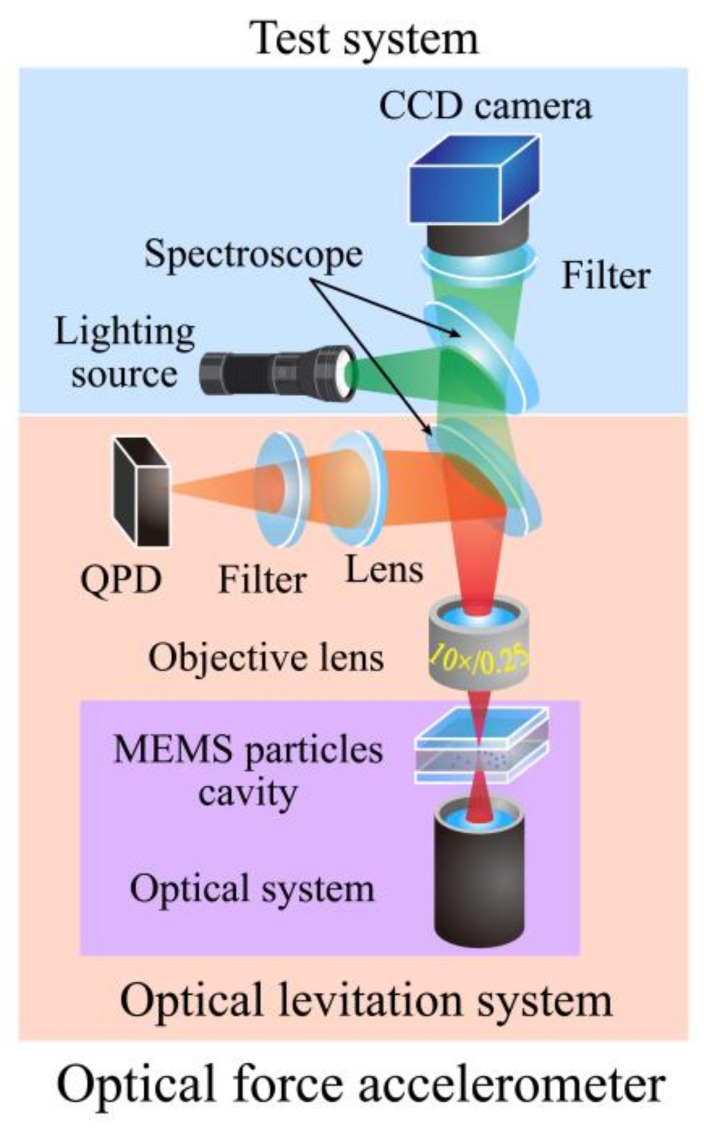
Optical path of the accelerometer and test system.

After constructing the optical levitation system, we determine the optical path structure of the miniature optical force accelerometer and the external test system, as shown in Figure 6.

### 3.1. Levitation Performance Test

**Figure 7:**
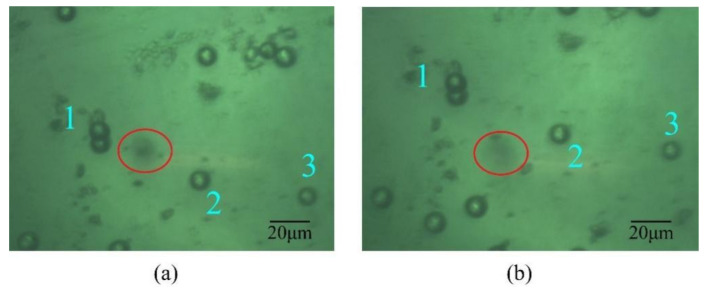
Trapping process of one 10 μm-diameter SiO_2_ in air. (**a**) The silica is trapped stably; (**b**) Process of the micro-particle’s cavity moving upward, particles 1, 2, 3 are used as a control reference.

After the laser focal plane, the imaging plane, and the bottom of the cavity are aligned, the vibration frequency of the piezoelectric sheet is adjusted to 100 kHz to excite the particles. When there is a blurred image of one particle in the field of vision, it suggests that the particle overcomes the van der Waals force and is trapped stably. Due to the good transparency of silica, we mainly use it as the trapping particle. Limited by the stability of laser power and the influence of van der Waals force on the particle, the diameter of the trapped particle can cover 8 μm to 13 μm when the laser power is adjusted from 115 mW to 140 mW. Due to the optical trap stiffness being related to laser power and particle size, unstable oscillation motion may occur when a single particle is levitated. Therefore, it is necessary to properly adjust the laser power and use the displacement platform to quickly and greatly adjust the particle’s cavity to ensure that the particle can still be firmly bound in the optical trap domain under large air resistance. Figure 7 shows the trapping process of one 10 μm-diameter SiO_2_ in air.

### 3.2. Experimental Test Setup of the Acceleration Test System

**Figure 8:**
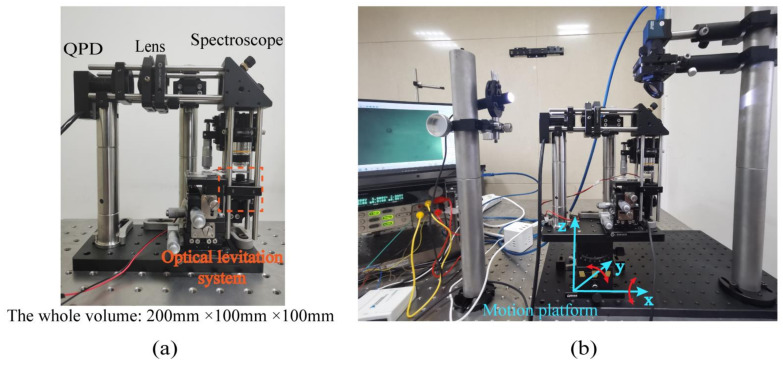
Photographs of the (**a**) accelerometer and the (**b**) test system.

When a silica particle is stably trapped by the optical levitation system, the divergent laser passes through the objective lens, and after the spectroscope, it is focused by a lens again and received by QPD. The above part also constitutes the whole of the optical force accelerometer. The external test system includes one lighting source, one spectroscope, and a charge-coupled device (CCD) with an infrared filter. This part is just used to determine whether one particle is captured by the optical trap in preparation. Photographs of the optical force accelerometer and the test system are shown in Figure 8. The physical and optical parameters of each component of the accelerometer are shown in Table 1.

The whole optical path is locked on a rotation platform whose size is 200 mm × 100 mm × 13 mm. This rotation platform has two degrees of freedom and can rotate around the x and y axes. When the platform is horizontal, the space angle is 0°. Its rotation range and resolution are, respectively, ±30° and 1°.

## 4. Test and Analysis of Optical Force Accelerometer

**Figure 9:**
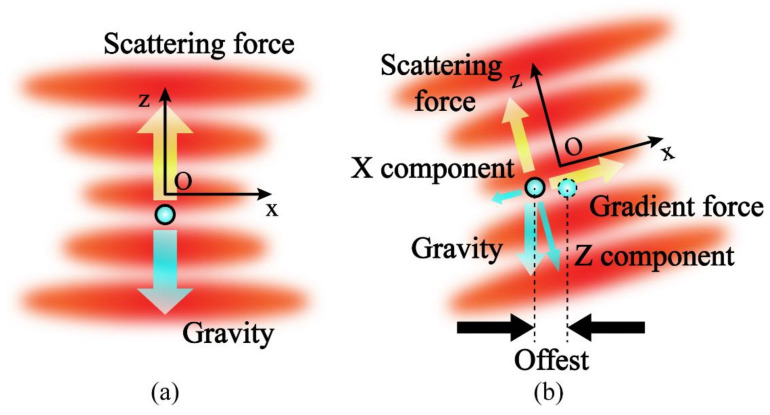
Principle of the acceleration calibration. (**a**) The optical trap direction is vertical upward; (**b**) The optical trap direction is rotated 15° around the y axis.

As the principle of this accelerometer mentioned in Section 2, under the action of the gravity component, as shown in Figure 9, the particle will deviate from the stable position after rotating the platform around one axis, and the offset will increase with the increase in dip angle [27]. Therefore, in the test, we use this way to calibrate the acceleration.

### 4.1. Calibration of Scale Factor

**Figure 10:**
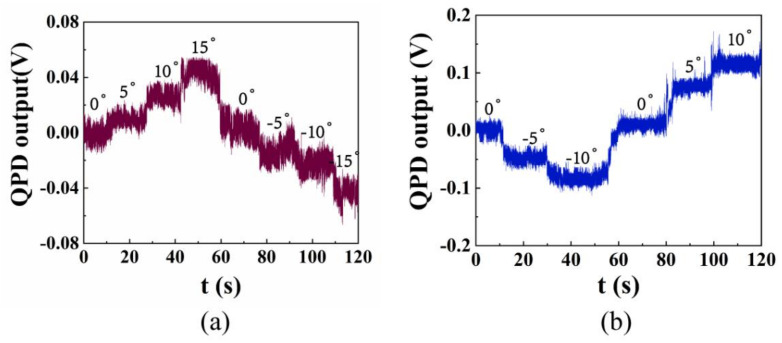
Signal of the optical force accelerometer. Variation of the output signal with the angle of platform changes along (**a**) the short axis and (**b**) the long axis of the elliptical beam.

Different from the standard Gaussian beam, the optical field distribution of the elliptical beam emitted by a laser diode is not consistent in the direction of the long axis and the short axis [28]. So it is necessary to calibrate the response of each sensing axis to the external acceleration. First, we test the acceleration response of the system by using one SiO_2_ with a diameter of 10 μm. The output signal is received by QPD when the platform rotates every five degrees and it is shown in Figure 10 where the unit of the output signal is volt.

As can be seen from Figure 10, on the premise of ensuring the stable capture of one particle, the rotation range (−15–15°) of the accelerometer in the direction of the short axis is significantly larger than that (−10–10°) in the direction of the extension axis, and the displacement deviation of the particle is smaller when the rotation angle is the same. This is because the gradient of the elliptic optical field is larger in the short axis direction, which leads to the greater stiffness of the optical trap in this direction. Due to our rotating platform adopting manual angle adjustment, there is obvious mechanical noise at the connection of each step.

However, when the rotation angle of the platform exceeds the above range, the optical trap stiffness will not be enough to overcome the gravity component of the particle. At this time, the particle will break away from the optical trap and fall to the bottom of the cavity.

**Figure 11:**
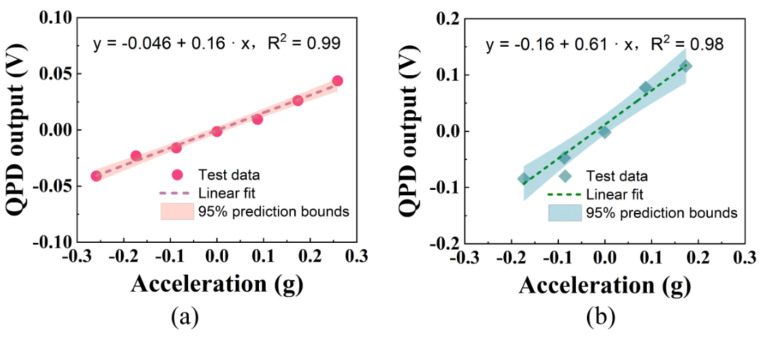
The fitting curve of the acceleration response along (**a**) the short axis from −15° to 15° and (**b**) the long axis from −10° to 10° of the elliptical beam.

The sine component of the acceleration of gravity at different angles is taken as the abscissa, the output signal of QPD is taken as the ordinate, the acceleration response curve can be obtained in Figure 11.

According to the consequence of linear fitting, the scale factor of the accelerometer is 0.16 V/g and 0.61 V/g along the short and long axes respectively. It also shows that there are differences in the acceleration response of the optical force accelerometer to different axes.

When the rotating platform is inclined by 10° along the long axis or inclined by 15° along the short axis, the particle will escape from the optical trap and fall onto the glass sheet under the action of gravity. Therefore, we believe that the range of the accelerometer should be g·sin10°-g·sin15° (±0.17 g–±0.26 g). This is affected by many factors, such as particle diameter, optical trap stiffness, acceleration input direction, and so on. The measurement resolution can be calculated from the acquisition voltage resolution and the voltage-acceleration scale factor. Due to the measuring range of the digital acquisition card that we selected being 5 V and the number of acquisition bits of this acquisition card being 14, its voltage resolution is 0.3 mV (5 V/2^14^).

After calculation, the measurement resolution of the short axis and the long axis is about 1.88 mg and 0.49 mg. The measurement resolution is mainly limited by the focusing quality of the light source, noise, and measurement accuracy of the photodetector.

### 4.2. Test of Stability in a Short Time

For accelerometers, they can be evaluated by measuring range, sensitivity, shock resistance, and other indexes, but the zero-bias stability is often the key performance that restricts its working environment. To characterize the short-term stability of the accelerometer, we use Allan variance to analyze it. Similar to the evaluation methods of variance and standard deviation in statistics, Allan variance is also a method to evaluate data, but it is only suitable for the evaluation of unstable random variables and the error term of various data [29].

The calculation process is shown as follows:

N data are divided into *K* (*K* = N/*n*) groups, each group contains *n* data points (generally *n* = 2j, j = 0, 1, 2, ...), and the average value of each group is:

(1) $$\bar{w_{k}}(n)=\frac{1}{n}\overset{n}{\underset{i=1}{\sum }}w_{\left(k-1\right)n+i}$$

(2) $$\begin{array}{c} \sigma_{A}^{2}\left(T\right)=\frac{1}{2}<{\left[{\bar{w}}_{k+1}\left(n\right)-\bar{w}\left(n\right)\right]}^{2}>\\ ≅\frac{1}{2\left(K+1\right)}\overset{K-1}{\underset{k=1}{\sum }}{\left[{\bar{w}}_{k+1}\left(n\right)-\bar{w}\left(n\right)\right]}^{2} \end{array}$$

**Figure 12:**
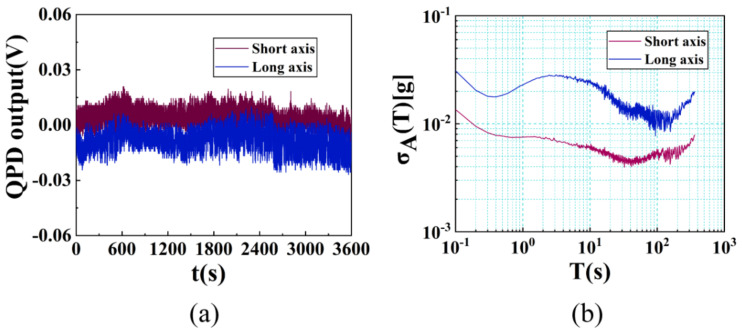
Test of the stability in an hour. (**a**) Original signal of the QPD; (**b**) Allan standard deviation of the accelerometer without external input.

After turning the angle of the platform to 0°, the stability of the accelerometer without input signal in an hour is tested. The sampling frequency is set as 10 Hz and the original signal is shown in Figure 12. After calculating the square root of Allan variance, we can evaluate the zero-bias stability of the system as about 4.4 mg and 9.2 mg along the short and long axes. It can be seen from the figure that although the curve of σ_A_ has obvious fluctuation, including the noise term with a slope of ±1/2, its fluctuation range is very small.

## 5. Discussion

From the original signal, it is not difficult to find that the output signal of QPD has a certain drift with the increase in time. This is because the temperature of the laser diode chip increases gradually with time, and the heat in the MEMS cavity also increases gradually. It not only affects the stiffness of the optical trap but also affects its stability. Due to the inhomogeneity of the laser diode optical field, the optical force accelerometer can better distinguish the acceleration input at two degrees of freedom, which is important for the improvement of the sensor performance. This may provide a new idea for the development of optical force accelerometers in the future.

According to different experimental requirements, the laser diode, photodetector and other devices of the accelerometer may be replaced with different models. After each replacement of any component, in addition to strictly aligning the long and short axes of the elliptical spot with the detector, the scale factor and other performance need to be tested again.

However, the proposed optical force accelerometer also has some limitations. Compared with the accelerometers with a high vacuum cavity, the noise of this accelerometer is larger. Due to the low stiffness of the optical trap, its ability to resist external environmental interference is also weak. Compared with other optical force accelerometers whose zero-bias stability is μg or even ng, the performance of this accelerometer needs to be improved. Moreover, in order to minimize the volume of the light source, we only use air cooling, which will lead to the heat generated by the diode after working for a long time, which will seriously affect the performance of the accelerometer. Therefore, improving the performance of the accelerometer under long-time operation is the focus of our next work.

## 6. Conclusions

In conclusion, a new form of optical accelerometer was developed in our work. By using an optical system composed of one laser diode, a pair of micro-lenses, and a particles chamber fabricated by MEMS technology, the packaging volume of the accelerometer is reduced to 200 mm × 100 mm × 100 mm. The measurement of acceleration was successfully realized by using this miniature optical force accelerometer. We calibrated and evaluated the response of the accelerometer to external acceleration by analyzing the offset signals exported by silicon particles with a diameter of 10 μm at different rotation angles. The experimental results show that the optical force accelerometer based on an elliptical beam has a different sensitivity to two orthogonal axial input signals. This property can also help it to be more sensitive to acceleration inputs in different directions. The Allan variance model is used to evaluate the short-term zero-bias stability of this accelerometer, which is about 4.4 mg and 9.2 mg in one hour. Although its measurement resolution (1.88 mg and 0.49 mg) can not reach the level of many other optical force accelerometers, it can measure acceleration with such a small volume and provide a miniaturized design idea. In order to further improve the performance of the optical force accelerometer, the detection optical path can be improved in the future, and the photoelectric detector with higher precision can be used to replace QPD, such as the balanced photodetector. Therefore, this new form of optical force accelerometer has a great potential to be an inertial sensor and it may provide a new choice for compact engineering devices in the future.

Future research should also consider the potential effects of the shape of the elliptical distribution of the Gaussian beam more carefully, for example, the optical trap stiffness can be controlled by changing the ellipticity of the optical field, to increase or reduce the sensitivity of the optical force accelerometer to signals in different directions. Moreover, other performance tests including the sensitivity of the optical force dual-axis accelerometer change with frequency may also constitute future work.

## Figures and Tables

**Table 1:** Physical and optical parameters of the accelerometer.

Component	Physical Size	Optical Parameter	
Laser diode	Φ 5.6 mm × 10 mm	Wavelength	808 nm
		Power	150 mW
Objective lens	Φ 22 mm × 38 mm	Magnification	10
Spectroscope	Φ 25.4 mm	Spectral ratio	50:50
Lens	Φ 25.4 mm	Focal length	20 mm
Filter	Φ 25.4 mm	Transmittance	1%

## Data Availability

Data underlying the results presented in this paper are not publicly available at this time but may be obtained from the authors upon reasonable request.
